# Blood pressure and lipid profiles in children born after ART with frozen embryo transfer

**DOI:** 10.1093/hropen/hoae016

**Published:** 2024-03-20

**Authors:** Louise Laub Asserhøj, Ikram Mizrak, Anna Sophie Lebech Kjaer, Tine Dalsgaard Clausen, Eva R Hoffmann, Gorm Greisen, Katharina M Main, Per Lav Madsen, Anja Pinborg, Rikke Beck Jensen

**Affiliations:** The Fertility Clinic, Department of Gynecology, Fertility and Obstetrics, Centre JMC, Copenhagen University Hospital—Rigshospitalet, Copenhagen, Denmark; Department of Growth and Reproduction, Copenhagen University Hospital—Rigshospitalet, Copenhagen, Denmark; International Centre for Research & Training in Disruption of Male Reproduction & Child Health (EDMaRC), Copenhagen University Hospital—Rigshospitalet, Copenhagen, Denmark; The Fertility Clinic, Department of Gynecology, Fertility and Obstetrics, Centre JMC, Copenhagen University Hospital—Rigshospitalet, Copenhagen, Denmark; Department of Cardiology, Copenhagen University Hospital—Herlev and Gentofte, Herlev, Denmark; The Fertility Clinic, Department of Gynecology, Fertility and Obstetrics, Centre JMC, Copenhagen University Hospital—Rigshospitalet, Copenhagen, Denmark; The Fertility Clinic, Department of Gynecology, Fertility and Obstetrics, Centre JMC, Copenhagen University Hospital—Rigshospitalet, Copenhagen, Denmark; Department of Clinical Medicine, University of Copenhagen, Copenhagen, Denmark; Department of Cellular and Molecular Medicine, Faculty of Health and Medical Sciences, Danish National Research Foundation (DNRF) Centre for Chromosome Stability, University of Copenhagen, Copenhagen, Denmark; Department of Clinical Medicine, University of Copenhagen, Copenhagen, Denmark; Department of Neonatology, Copenhagen University Hospital Rigshospitalet, Copenhagen, Denmark; Department of Growth and Reproduction, Copenhagen University Hospital—Rigshospitalet, Copenhagen, Denmark; International Centre for Research & Training in Disruption of Male Reproduction & Child Health (EDMaRC), Copenhagen University Hospital—Rigshospitalet, Copenhagen, Denmark; Department of Clinical Medicine, University of Copenhagen, Copenhagen, Denmark; Department of Cardiology, Copenhagen University Hospital—Herlev and Gentofte, Herlev, Denmark; Department of Clinical Medicine, University of Copenhagen, Copenhagen, Denmark; The Fertility Clinic, Department of Gynecology, Fertility and Obstetrics, Centre JMC, Copenhagen University Hospital—Rigshospitalet, Copenhagen, Denmark; Department of Clinical Medicine, University of Copenhagen, Copenhagen, Denmark; Department of Clinical Medicine, University of Copenhagen, Copenhagen, Denmark; Department of Paediatrics, Copenhagen University Hospital—Herlev and Gentofte, Herlev, Denmark

**Keywords:** ART, frozen embryo transfer, blood pressure, lipid profile, childhood

## Abstract

**STUDY QUESTION:**

Are blood pressure (BP) and lipid profiles different between children conceived after ART with frozen embryo transfer (FET), fresh embryo transfer (fresh-ET), and natural conception (NC)?

**SUMMARY ANSWER:**

Girls conceived after FET had significantly higher systolic BP and heart rate compared with girls born after fresh-ET; boys conceived after FET had a slightly more favourable lipid profile compared with boys born after fresh-ET and NC.

**WHAT IS KNOWN ALREADY:**

Children conceived after ART with FET are more often born large for gestational age (LGA). LGA in general increases the risk of obesity, diabetes, and cardiovascular disease later in life. Studies on mice and humans on the whole ART population have raised concerns about premature vascular ageing and higher BP. The cardiovascular health of children born after FET is scarcely explored and the results are diverging.

**STUDY DESIGN, SIZE, DURATION:**

This study was part of the cohort study ‘Health in Childhood following Assisted Reproductive Technology’ (HiCART), which included 606 singletons (292 boys) born between December 2009 and December 2013: 200 children were conceived after FET; 203 children were conceived after fresh-ET; and 203 children were conceived naturally and matched for birth year and sex. The study period lasted from January 2019 to September 2021.

**PARTICIPANTS/MATERIALS, SETTING, METHODS:**

The included children were 7–10 years of age at examination and underwent a clinical examination with anthropometric measurements, pubertal staging, and BP measurement. Additionally, a fasting blood sample was collected and analysed for cholesterol, low-density lipoproteins (LDL), high-density lipoproteins (HDL), and triglycerides. Systolic and diastolic BP were converted to standard deviation scores (SDS) using an appropriate reference and accounting for height (SDS) of the child. The three study groups were compared pairwise using a univariate linear regression model. Mean differences were adjusted for confounders using multiple linear regression analyses.

**MAIN RESULTS AND THE ROLE OF CHANCE:**

Girls and boys conceived after FET had significantly higher birthweight (SDS) compared with naturally conceived peers (mean difference: girls: 0.35, 95% CI (0.06–0.64), boys: 0.35, 95% CI (0.03–0.68)). Girls conceived after FET had significantly higher systolic BP (SDS) and heart rate compared with girls conceived after fresh-ET (adjusted mean difference: systolic BP (SDS): 0.25 SDS, 95% CI (0.03–0.47), heart rate: 4.53, 95% CI (0.94–8.13)). Regarding lipid profile, no significant differences were found between the three groups of girls. For the boys, no significant differences were found for BP and heart rate. Lipid profiles were more favourable in boys born after FET compared with both boys conceived after fresh-ET and NC. All outcomes were adjusted for parity, maternal BMI at early pregnancy, smoking during pregnancy, educational level, birthweight, breastfeeding, child age at examination, and onset of puberty.

**LIMITATIONS, REASONS FOR CAUTION:**

The participation rate varied from 18 to 42% in the three groups, and therefore selection bias cannot be excluded. However, extensive non-participant analyses were performed that showed almost no differences in background characteristics between participants and non-participants in the three groups, making selection bias less likely.

**WIDER IMPLICATIONS OF THE FINDINGS:**

The higher birthweight in children conceived after FET was associated with increased systolic BP (SDS) and heart rate in girls conceived after FET compared with fresh-ET. This may be an early indicator of compromised long-term cardiovascular health in this group. The study was not powered to investigate these outcomes and further studies are therefore warranted to confirm the findings.

**STUDY FUNDING/COMPETING INTEREST(S):**

The study was funded by the Novo Nordisk Foundation (grant number: NNF18OC0034092, NFF19OC0054340) and Rigshospitalets Forskningsfond. The authors have no conflicts of interest to declare.

**TRIAL REGISTRATION NUMBER:**

ClinicalTrials.gov identifier: NCT03719703.

WHAT DOES THIS MEAN FOR PATIENTS? More than 10 million children have been born after the use of assisted reproductive technology (ART). Especially the use of frozen, and later on thawed, embryos has been increasing steadily during the last decade. The health of the children born after ART is of utmost interest to the parents and to society. Previous studies have shown that children born after the use of frozen/thawed embryos are born with a higher birthweight compared with children conceived naturally or after the use of fresh embryos. However, the potential long-term implications of this elevated birthweight remain insufficiently explored. This study investigated the blood pressure and the lipid profile (e.g. blood cholesterol levels) of 606 Danish children aged 7–10 years born after the use of frozen/thawed embryos, fresh embryos, and natural conception. Reassuringly, our results reveal no differences between the three study groups. When examining the girls and boys separately, small differences emerge, which need to be explored further.

## Introduction

Animal and human studies have led to concern about the cardiometabolic health in offspring conceived after ART (Guo *et al.*, 2017; Cui *et al.*, 2020; Elhakeem *et al.*, 2023). Mice bred by IVF presented altered fatty-acid metabolism (Wang *et al.*, 2013), endothelial dysfunction, increased vascular stiffness (Rexhaj *et al.*, 2013), and elevated systolic blood pressure (SBP) (Watkins *et al.*, 2007). However, results from human studies are diverging. A meta-analysis from 2017 showed that children born after ART had significantly higher SBP and diastolic blood pressure (DBP), a suboptimal diastolic function, and increased vessel thickness but lower low-density lipoprotein (LDL) compared with children born after natural conception (NC) (Guo *et al.*, 2017). On the other hand, a study from 2023 included data from 14 birth cohorts and found no differences in SBP and DBP but increased total cholesterol, high-density lipoprotein (HDL) and LDL in children conceived after ART compared with NC (Elhakeem *et al.*, 2023). Further, two Australian studies found similar or favourable blood pressure (BP), lipid profiles, and vascular structures in adolescents and young adults born after ART compared with NC (Halliday *et al.*, 2019; Wijs *et al.*, 2022).

Until the late 2000s, the majority of ART-children were born after fresh embryo transfer (fresh-ET) but after the introduction of vitrification, frozen embryo transfer (FET) today accounts for 37% of the ART cycles in Europe (Smeenk *et al.*, 2023) and more than 70% of ART cycles in the USA (Centers for Disease Control and Prevention, 2023). FET has clear benefits by facilitating single embryo transfers and freeze-all strategies, reducing the risk of multiple pregnancies and reducing the incidence of ovarian hyperstimulation syndrome (OHSS) owing to the avoidance of a fresh embryo transfer in women with high risk of OHSS. However, studies indicate that children born after FET, especially those born after FET cycles with hormone replacement therapy (HRT-FET) are more prone to adverse obstetric and neonatal outcomes, such as hypertensive disorders in pregnancy, pre-eclampsia, post-partum haemorrhage, caesarean section, and high birthweight (Asserhøj *et al.*, 2021; Moreno-Sepulveda *et al.*, 2021; Rosalik *et al.*, 2021; Busnelli *et al.*, 2022), compared with children born after FET in a natural cycle (NC-FET). Further, children born after FET have an increased risk of being born with a high birthweight and large for gestational age (LGA) compared with children born after both fresh-ET and NC (Berntsen and Pinborg, 2018; Terho *et al.*, 2021; Westvik-Johari *et al.*, 2021), which may pose an elevated risk of adverse cardiometabolic outcomes later in life (Zhang *et al.*, 2023).

Studies of long-term cardiovascular outcomes of children born after FET are limited, performed on heterogeneous cohorts, and the results diverge. The first study in the area included 65 ART-children (17 of them born after FET) and found generalized vascular dysfunction in ART-children compared with children conceived naturally persisting through adolescence, but with no differences between FET and fresh-ET (Scherrer *et al.*, 2012; Meister *et al.*, 2018). Another study found no differences in lipid profiles, except for lower HDL, in children born after FET compared with NC (Green *et al.*, 2013). A register study reported similar risk of cardiovascular disease when comparing FET with fresh-ET (Norrman *et al.*, 2021). The multi-cohort study by Elhakeem *et al.* (2023) conducted a sub-analysis including only two cohorts and compared FET and fresh-ET with NC and found similar results in terms of SBP, DBP, heart rate, and lipid profile, with the exception of higher total cholesterol levels in the FET group compared with the NC group. A recently published study observed a higher SBP in 569 children born after FET compared with 396 children born after fresh-ET, but only in the subgroup with overweight mothers (Zhang *et al.*, 2022).

Our previous findings showed increased height, weight, hip- and waist circumference in girls but not boys born after FET (Asserhøj *et al.*, 2023) and, therefore, the aim of this study was to explore differences in BP, heart rate, and lipid profiles in a sex-stratified cohort of children aged 7–10 years born after FET, fresh-ET, or NC.

## Materials and methods

### Study design and study population

The ‘Health in Childhood following Assisted Reproductive Technology’ (HiCART) cohort consists of 606 singletons (314 girls and 292 boys) aged 7–10 years, born from November 2009 to December 2013 in the Eastern part of Denmark. The cohort has previously been described in detail (Asserhøj *et al.*, 2023). In brief, the singletons were identified through the Danish Medical Birth Registry and the Danish IVF Registry and were recruited into three groups according to mode of conception: ART with FET (n = 200), ART with fresh-ET (n = 203), and naturally conceived (n = 203) and matched for sex and year of birth.

A total of 2314 mothers and their children were invited to take part in the study. Of them, 606 children were included as participants. The participation rate across all groups was 26%, however with variation between the three study groups (FET: 42%, fresh-ET: 31%, NC: 18%) (previously described in detail (Asserhøj *et al.*, 2023)). Additionally, 36 mothers who had initially responded positively to the invitation were excluded based on the exclusion criteria (mothers with diabetes mellitus or gestational diabetes, twins, siblings, children born after oocyte donation, and children with severe illnesses (as assessed by a specialized paediatrician)).

All children in the FET group were conceived by embryos frozen with the slow–freeze method (9% missing data). Regarding day of transfer, 67% had a cleavage-stage embryo transferred in the FET group; blastocyst transfer accounted for 4%; and in 29% of the children, information on culture time was missing. In the fresh-ET group, 86% had a cleavage-stage embryo transferred (6% blastocyst, 8% missing data). Further, HRT-FET was utilized in 13.5% of the pregnancies after FET as endometrial preparation; in 10.5%, true natural cycle-FET was used; and modified natural cycle-FET accounted for 57% of the FET cycles. In 19% of FET pregnancies, data on type of FET protocol were unavailable.

### Outcome measures

All clinical examinations were performed at the Department of Growth and Reproduction, Rigshospitalet, Copenhagen, and all examiners were blinded for mode of conception of the child.

### Blood pressure

During the clinical examination, BP and heart rate of the child were measured non-invasively after 5 min of rest in a supine position with a cuff appropriately sized to fit the child’s arm (Criticare Technologies, Comfortcuff 5063N3 Series, Warwick, RI, USA). Three sequential measurements with 2-min intervals were performed and an average was used. SBP and DBP were converted into standard deviation scores (SDS) according to sex, age, and height using a mixed effect linear regression model and an American child cohort as the reference population (Falkner and Daniels, 2004).

### Puberty assessment

A trained clinician performed pubertal staging according to Marshall and Tanner (1969, 1970). In females, the onset of puberty was defined as breast stage ≥2, while in males, testicular volume exceeding 3 ml (measured using a Prader orchidometer) indicated the beginning of puberty.

### Biochemical measurements

A blood sample was drawn from an antecubital vein after a minimum 8 h of fasting. Blood samples were available from 577 out of 606 children (95.2%). The lipid profiles (cholesterol, LDL, HDL, and triglyceride) were analysed with enzymatic calorimetric analysis (Cobas 8000, c702 system; Abell/Kendall, Roche Diagnostics GmbH, Mannheim, Germany).

### Statistical analysis

R for Windows (version 3.6.1, a language and environment for statistical computing, Vienna, Austria) and the Rstudio platform (version 1.2.5001) were utilized to conduct all statistical analyses. To ensure independence within the groups, siblings were excluded.

One participant (a boy from the FET group) was removed from the analyses as an outlier owing to systolic BP >4 SD. He had a known diagnosis of attention-deficit hyperactivity disorder and was currently treated with methylphenidate, which has the known side effect of hypertension. The data are displayed as mean (SD) and number (%), as appropriate.

For background characteristics and cardiovascular measurements, pairwise comparisons were made (FET versus fresh-ET, FET versus NC, and fresh-ET versus NC). Univariate linear regression analysis was utilized for continuous variables and mean differences are reported. For categorical variables, a two-sided Chi-square test was used, and risk differences are shown. With the use of multiple linear regression analyses, all outcome measurements were adjusted for possible confounders (parity, maternal BMI at early pregnancy, maternal smoking during pregnancy, maternal educational level, birthweight, breastfeeding, child age at examination, and onset of puberty (yes/no)). The background characteristics and the results of the study are presented stratified by sex.

A thorough non-participant analysis of the entire cohort was conducted and has been described in detail in a previous publication (Asserhøj *et al.*, 2023).

### Ethics approval

Following the guidelines of the Declaration of Helsinki, written informed consent was obtained from all parents who had full custody of the child. The parents were provided with both written and verbal information about the project before giving their consent. The study protocol was granted approval by the Ethics Committee of the Capital Region of Denmark (H-18000122) and The Danish Data Protection Agency (VD-2018-310).

## Results

### Background characteristics

Girls and boys born after FET both had significantly higher birthweight (SDS) compared with NC (mean difference: girls: 0.35, 95% CI (0.06 to 0.64), boys: 0.35, 95% CI (0.03 to 0.68)). Maternal and paternal age at birth was higher in the two ART groups compared with NC for both sexes. Further, parity was lower in the FET group compared with fresh-ET and NC for both sexes. The paternal educational level was higher in boys born after FET than in the boys born after NC. Mothers of the boys in the two ART groups were less likely to smoke during pregnancy compared with boys in the NC group; however, these findings were not seen for the girls. We observed no significant differences between the three groups regarding gestational age, maternal BMI in early pregnancy and at examination, and parental self-estimated health (Tables 1 and 2).

**Table 1.** Basic characteristics of the girls included in the study divided into the three study groups and compared pairwise.

	FET		Fresh-ET		NC		FET vs fresh-ET^a^	FET vs NC^a^	Fresh-ET vs NC^a^
	n		n		n				
**Child characteristics**									
Gestational age, days^b^	112	278 (12.1)	101	280 (9.4)	99	279 (9.1)	–1.68 (–4.62 to 1.26)	–1.22 (–4.15 to 1.71)	0.46 (–2.11 to 3.03)
Birthweight, g^b^	112	3,531 (496)	101	3,470 (434)	99	3,436 (475)	61.15 (–65.35 to 187.65)	95.17 (–37.09 to 227.43)	34.02 (–92.77 to 160.81)
Birthweight, SDS^b^	112	0.19 (1.05)	101	–0.08 (1.04)	98	–0.16 (1.08)	0.27 (–0.02 to 0.55)	**0.35 (0.06 to 0.64)**	0.08 (–0.22 to 0.38)
Birthweight for gestational age, SDS (n (%))	112		101		98				
SGA (<–2SD)		3 (2.7)		5 (5.0)		4 (4.1)	–0.02 (–0.08 to 0.04)	–0.01 (–0.07 to 0.04)	0.01 (–0.06 to 0.08)
AGA		104 (92.9)		95 (94.1)		92 (93.9)	–0.02 (–0.09 to 0.06)	–0.01 (–0.09 to 0.07)	0.00 (–0.07 to 0.07)
LGA (>2SD)		5 (4.5)		1 (1.0)		2 (2.0)	0.03 (–0.02 to 0.09)	0.03 (–0.03 to 0.08)	–0.01 (–0.05 to 0.03)
**Maternal characteristics**									
Age at delivery, years ^b^	106	35.3 (4.5)	95	34.4 (4.5)	97	32.4 (4.6)	0.85 (–0.40 to 2.10)	**2.85 (1.59 to 4.11)**	**1.99 (0.71 to 3.28)**
Fertilization (n (%))^b^	98		98		101				
IVF		66 (67.3)		56 (57.1)		0 (0.0)	0.10 (–0.04 to 0.25)	–	–
ICSI		32 (32.7)		42 (42.9)		0 (0.0)	–0.10 (–0.25 to 0.04)	–	–
Spontaneously		0 (0.0)		0 (0.0)		101 (100)	–	–	–
Parity, (n (%))^b^	112		100		100				
Nulliparous		60 (53.6)		78 (78.0)		44 (44.0)	–**0.24 (**–**0.37 to** –**0.11)**	0.14 (–0.00 to 0.27)	**0.38 (0.25 to 0.51)**
BMI at early pregnancy, kg/m^2^ (n (%))^b^	112		100		96				
<18.5		2 (1.8)		2 (2.0)		4 (4.2)	–0.00 (–0.04 to 0.04)	–0.02 (–0.08 to 0.03)	–0.02(–0.08 to 0.04)
18.5–24.9		85 (75.9)		74 (74.0)		67 (69.8)	0.02 (–0.11 to 0.15)	0.06 (–0.07 to 0.19)	0.04 (–0.09 to 0.18)
≥25.0		25 (22.3)		24 (24.0)		25 (26.0)	–0.02 (–0.14 to 0.11)	–0.04 (–0.16 to 0.09)	–0.02 (–0.15 to 0.11)
Smoking during pregnancy (n (%))^b^	112		101		101				
Yes		2 (1.8)		1 (1.0)		6 (5.9)	0.01 (–0.03 to 0.05)	–0.04 (–0.10 to 0.02)	–0.05 (–0.11 to 0.01)
Solely breastfeeding (month) (n (%))^c^	95		82		85				
0		7 (7.4)		7 (8.5)		3 (3.5)	–0.01 (–0.10 to 0.08)	0.04 (–0.04 to 0.12)	0.05 (–0.03 to 0.13)
>0–4		27 (28.4)		25 (30.1)		36 (42.4)	–0.02 (–0.17 to 0.13)	–0.14 (–0.29 to 0.01)	–0.12 (–0.28 to 0.04)
>4		61 (64.2)		50 (61.0)		46 (54.1)	0.03 (–0.12 to 0.19)	0.10 (–0.05 to 0.26)	0.07 (–0.09 to 0.23)
BMI at examination of child (kg/m^2^) (n (%))^c^	106		95		97				
<18.5		0 (0.0)		1 (1.1)		0 (0.0)	–0.01 (–0.04 to 0.02)	–	0.01 (–0.02 to 0.04)
18.5–24.9		71 (67.0)		62 (65.3)		59 (60.8)	0.02 (–0.12 to 0.16)	0.06 (–0.08 to 0.20)	0.04 (–0.10 to 0.19)
≥25.0		35 (33.0)		32 (33.7)		38 (39.2)	–0.01 (–0.14 to 0.13)	–0.06 (–0.20 to 0.08)	–0.05 (–0.20 to 0.09)
Self-estimated health (n (%))^c^	104		95		97				
Excellent/very good		74 (71.2)		66 (69.5)		73 (75.3)	0.02 (–0.12 to 0.15)	–0.04 (–0.17 to 0.09)	–0.07 (–0.19 to 0.08)
Good		23 (22.1)		27 (28.4)		22 (22.7)	–0.06 (–0.19 to 0.07)	–0.01 (–0.13 to 0.12)	0.06 (–0.08 to 0.19)
Poor		7 (6.7)		2 (2.1)		2 (2.1)	0.05 (–0.02 to 0.11)	0.05 (–0.02 to 0.11)	0.00 (–0.04 to 0.04)
Educational level (n (%))^c^	104		95		97				
Higher education >4 years		37 (35.6)		40 (42.1)		35 (36.1)	–0.07 (–0.21 to 0.08)	–0.01 (–0.14 to 0.13)	0.06 (–0.09 to 0.21)
**Paternal characteristics**									
Age at delivery, years^c^	90	37.4 (5.5)	80	36.7 (5.9)	87	34.1 (6.0)	0.69 (–1.04 to 2.42)	**3.32 (1.61 to 5.03)**	**2.63 (0.82 to 4.45)**
BMI at examination of child, kg/m^2^ (n (%))^c^	90		83		88				
<18.5		0 (0.0)		1 (1.2)		0 (0.0)	–0.01 (–0.05 to 0.02)	–	0.01 (–0.02 to 0.05)
18.5–24.9		40 (44.4)		44 (53.0)		33 (37.5)	–0.09 (–0.25 to 0.07)	0.07 (–0.09 to 0.22)	0.16 (–0.00 to 0.31)
≥25.0		50 (55.6)		38 (45.8)		55 (62.5)	0.10 (–0.06 to 0.26)	–0.07 (–0.22 to 0.09)	–**0.17 (**–**0.33to** –**0.01)**
Self-estimated health (n (%))^c^	90		83		88				
Excellent/very good		60 (66.7)		54 (65.1)		51 (58.0)	0.02 (–0.14 to 0.17)	0.09 (–0.07 to 0.24)	0.07 (–0.09 to 0.23)
Good		27 (30.0)		28 (33.7)		36 (40.9)	–0.04 (–0.19 to 0.11)	–0.11 (–0.26 to 0.04)	–0.07 (–0.23 to 0.08)
Poor		3 (3.3)		1 (1.2)		1 (1.1)	0.02 (–0.03 to 0.08)	0.02 (–0.03 to 0.08)	0.00 (–0.03 to 0.03)
Educational level (n (%))^c^	90		83		88				
Higher education >4 years		37 (41.1)		31 (37.3)		34 (38.6)	0.04 (–0.12 to 0.19)	0.02 (–0.14 to 0.18)	–0.01 (–0.17 to 0.14)

Values are shown as mean (SD).

a Pairwise comparison of groups by calculating mean differences with 95% CI for continuous variables (using univariate linear regression model) and risk differences with 95% CI for categorical variables (using a two-sided Chi-square-test).

b Information from the Danish National IVF registry.

c Information from questionnaire.

AGA: appropriate for gestational age; FET: frozen embryo transfer; fresh-ET: fresh embryo transfer; LGA: large for gestational age; NC: natural conception; SDS: standard deviation score; SGA: small for gestational age.

Bold text indicates significant differences between the study groups investigated.

**Table 2.** Basic characteristics of the boys included in the study divided into the three study groups and compared pairwise.

	FET		Fresh-ET		NC		FET vs fresh-ET^a^	FET vs NC^a^	Fresh-ET vs NC^a^
	n		n		n				
**Child characteristics**									
Gestational age, days^b^	87	279 (12.2)	102	278 (12.4)	100	278 (12.4)	0.40 (–3.14 to 3.93)	0.81 (–2.74 to 4.36)	0.41 (–3.02 to 3.85)
Birthweight, g^b^	87	3670 (545)	102	3434 (528)	101	3505 (520)	**236.95 (82.22 to 390.89)**	**165.23 (11.83 to 318.62)**	–71.32 (–216.35 to 73.70)
Birthweight, SDS^b^	87	0.20 (1.14)	102	–0.36 (0.95)	99	–0.15 (1.10)	**0.56 (0.26 to ** **0.86)**	**0.35 (0.03 to 0.68)**	–0.21 (–0.49 to 0.08)
Birthweight for gestational age, SDS (n (%))	87		102		99				
SGA (<–2SD)		2 (2.3)		4 (3.9)		3 (3.0)	–0.02 (–0.08 to 0.04)	–0.01 (–0.06 to 0.05)	0.01 (–0.05 to 0.07)
AGA		81 (93.1)		96 (94.1)		94 (94.9)	–0.01 (–0.09 to 0.07)	–0.02 (–0.10 to 0.06)	–0.01 (–0.08 to 0.06)
LGA (>2SD)		4 (4.6)		2 (2.0)		2 (2.0)	0.03 (–0.04 to 0.09)	0.03 (–0.04 to 0.09)	–0.00 (–0.04 to 0.04)
**Maternal characteristics**									
Age at delivery, years^b^	79	34.3 (3.6)	98	34.9 (4.5)	87	31.7 (5.6)	–0.55 (–1.79 to 0.69)	**2.59 (1.13 to 4.04)**	**3.14 (1.67 to 4.60)**
Fertilization (n (%))^b^	78		98		102				
IVF		51 (65.4)		52 (53.1)		0 (0.0)	0.12 (–0.03 to 0.28)	–	–
ICSI		27 (34.6)		46 (46.9)		0 (0.0)	–0.12 (–0.28 to 0.03)	–	–
Spontaneously		0 (0.0)		0 (0.0)		203 (100)	–	–	–
Parity (n (%))^b^	87		102		101				
Nulliparous		48 (55.2)		82 (80.1)		50 (49.5)	–**0.25 (**–**0.39to** –**0.11)**	0.06 (–0.10 to 0.21)	**0.31 (0.17 to 0.44)**
BMI at early pregnancy, kg/m^2^ (n (%))^b^	86		96		97				
<18.5		2 (2.3)		2 (2.1)		2 (2.1)	0.00 (–0.04 to 0.05)	0.00 (–0.04 to 0.05)	0.00 (–0.04 to 0.04)
18.5–24.9		68 (79.1)		68 (70.8)		67 (69.1)	0.08 (–0.05 to 0.22)	0.10 (–0.04 to 0.24)	0.02 (–0.12 to 0.16)
≥25.0		16 (18.6)		26 (27.1)		28 (28.9)	–0.08 (–0.22 to 0.05)	–0.10 (–0.24 to 0.03)	–0.02 (–0.15 to 0.12)
Smoking during pregnancy (n (%))^b^	86		102		99				
Yes		1 (1.2)		1 (1.0)		11 (11.1)	0.00 (–0.03 to 0.03)	–**0.10 (**–**0.18to **–**0.02)**	–**0.10 (**–**0.18to** –**0.03)**
Solely breastfeeding, month (n (%))^c^	73		87		78				
0		3 (4.1)		7 (8.0)		2 (2.6)	–0.05 (–0.13 to 0.03)	0.02 (–0.06 to 0.09)	0.05 (–0.02 to 0.13)
>0–4		26 (35.6)		26 (29.9)		37 (47.4)	0.06 (–0.10 to 0.22)	–0.12 (–0.29 to 0.05)	–**0.18 (**–**0.33to** –**0.02)**
>4		44 (60.3)		54 (62.1)		39 (50.0)	–0.02 (–0.18 to 0.15)	0.10 (–0.07 to 0.27)	0.12 (–0.04 to 0.28)
BMI at examination of child, kg/m^2^ (n (%))^c^	79		98		86				
<18.5		1 (1.3)		1 (1.0)		2 (2.3)	0.00 (–0.03 to 0.04)	–0.01 (–0.06 to 0.04)	–0.01 (–0.06 to 0.04)
18.5–24.9		54 (68.4)		57 (58.2)		55 (64.0)	0.10 (–0.05 to 0.25)	0.04 (–0.11 to 0.20)	–0.06 (–0.21 to 0.09)
≥25.0		24 (30.4)		40 (40.8)		29 (33.7)	–0.10 (–0.26 to 0.05)	–0.03 (–0.19 to 0.12)	0.07 (–0.08 to 0.22)
Self-estimated health (n (%))^c^	78		96		87				
Excellent/very good		49 (62.8)		58 (60.4)		61 (70.1)	0.02 (–0.13 to 0.18)	–0.07 (–0.23 to 0.08)	–0.10 (–0.25 to 0.05)
Good		27 (34.6)		37 (38.5)		22 (25.3)	–0.04 (–0.19 to 0.12)	0.09 (–0.06 to 0.25)	0.13 (–0.01 to 0.28)
Poor		2 (2.6)		1 (1.0)		4 (4.6)	0.02 (–0.04 to 0.07)	–0.02 (–0.09 to 0.05)	–0.04 (–0.09 to 0.02)
Educational level (n (%))^c^	78		96		87				
Higher education > 4 years		40 (51.3)		41 (42.7)		35 (40.2)	0.09 (–0.07 to 0.25)	0.11 (–0.05 to 0.27)	0.02 (–0.13 to 0.18)
**Paternal characteristics**									
Age at delivery, years^c^	65	37.9 (5.1)	72	37.3 (6.2)	70	33.6 (5.8)	0.62 (–1.31 to 2.55)	**4.31 (2.45 to 6.18)**	**3.69 (1.70 to 5.69)**
BMI at examination of child, kg/m^2^ (n (%))^c^	65		73		72				
<18.5		0 (0.0)		0 (0.0)		0 (0.0)	–	–	–
18.5–24.9		31 (47.7)		23 (31.5)		36 (50.0)	0.16 (–0.01 to 0.34)	–0.02 (–0.21 to 0.16)	–**0.19 (**–**0.36to** –**0.01)**
≥25.0		34 (52.3)		50 (68.5)		36 (50.0)	–0.16 (–0.34 to 0.01)	0.02 (–0.16 to 0.21)	**0.19 (0.01 to 0.36)**
Self-estimated health (n (%))^c^	65		73		72				
Excellent/very good		42 (64.6)		44 (60.3)		39 (54.2)	0.04 (–0.13 to 0.22)	0.10 (–0.07 to 0.28)	0.06 (–0.11 to 0.24)
Good		21 (32.3)		27 (37.0)		29 (40.3)	–0.05 (–0.22 to 0.13)	–0.08 (–0.25 to 0.10)	–0.03 (–0.21 to 0.14)
Poor		2 (3.1)		2 (2.7)		4 (5.6)	0.00 (–0.06 to 0.06)	–0.02 (–0.11 to 0.06)	–0.03 (–0.11 to 0.05)
Educational level (n (%))^c^	65		73		72				
Higher education >4 years		33 (50.8)		30 (41.1)		23 (31.9)	0.10 (–0.08 to 0.28)	**0.19 (0.01 to 0.37)**	0.09 (–0.08 to 0.26)

Values are shown in means (SD).

a Pairwise comparison of groups by calculating mean differences with 95% CI for continuous variables (using univariate linear regression model) and risk differences with 95% CI for categorical variables (using a two-sided Chi-square-test).

b Information from the Danish National IVF registry.

c Information from questionnaire.

AGA: appropriate for gestational age; FET: frozen embryo transfer; fresh-ET: fresh embryo transfer; LGA: large for gestational age; NC: natural conception; SDS: standard deviation score; SGA: small for gestational age.

Bold text indicates significant differences between the study groups investigated.

### BP and heart rate

Girls born after FET had significantly higher adjusted (adj.) SBP and SBP (SDS) compared with girls born after fresh-ET (adj. mean difference: SBP: 2.85, 95% CI (0.51 to 5.19), SBP (SDS); 0.25, 95% CI (0.03 to 0.47)) but not compared with girls born after NC. The DBP was similar in all three groups, both investigating the raw BP values and the data converted in to SDS. Further, girls born after FET had a significantly higher heart rate compared with girls born after fresh-ET (adj. mean difference: 4.53, 95% CI (0.94 to 8.13)), but no difference compared with NC (Table 3). For the boys, we found no significant differences in SBP, DBP, and heart rate between any of the three groups (Table 4).

**Table 3.** Cardiovascular outcomes for girls included in the study divided into the three study groups and compared pairwise.

	FET n = 112	Fresh-ET n = 101	NC n = 101	FET vs fresh-ET^a^ adjusted	FET vs NC^a^ adjusted	Fresh-ET vs NC^a^ adjusted
Age at examination, years	8.43 (0.56)	8.55 (0.50)	8.65 (0.57)	–0.10 (–0.27 to 0.06)	–**0.22 (**–**0.38to** –**0.05)**	–0.11 (–0.29 to 0.06)
Systolic blood pressure, mmHG	107.46 (9.06)	104.65 (6.70)	106.04 (6.41)	**2.85 (0.51 to 5.19)**	2.19 (–0.14 to 4.51)	–0.66 (–3.10 to 1.77)
Systolic blood pressure, SDS	0.75 (0.84)	0.49 (0.61)	0.61 (0.59)	**0.25 (0.03 to 0.47)**	0.18 (–0.04 to 0.39)	–0.07 (–0.30 to 0.16)
Diastolic blood pressure, mmHG	65.23 (6.22)	63.45 (6.30)	65.06 (5.92)	0.13 (–0.05 to 0.30)	0.04 (–0.12 to 0.22)	–0.08 (–0.26 to 0.10)
Diastolic blood pressure, SDS	0.54 (0.57)	0.38 (0.58)	0.52 (0.53)	0.13 (–0.05 to 0.30)	0.04 (–0.13 to 0.22)	–0.08 (–0.26 to 0.10)
Heart rate, bpm	83.46 (13.25)	79.68 (11.02)	80.95 (9.60)	**4.53 (0.94 to 8.13)**	0.94 (–2.64 to 4.52)	–3.59 (–7.33 to 0.14)
Cholesterol, mmol/L	4.12 (0.62)	4.10 (0.70)	4.02 (0.58)	0.07 (–0.12 to 0.26)	0.11 (–0.08 to 0.31)	0.04 (–0.16 to 0.25)
Low-density lipoprotein, mmol/L	2.40 (0.56)	2.43 (0.61)	2.33 (0.56)	0.04 (–0.15 to 0.22)	0.11 (–0.07 to 0.29)	0.07 (–0.12 to 0.26)
High-density lipoprotein, mmol/L	1.61 (0.33)	1.59 (0.33)	1.59 (0.30)	0.01 (–0.08 to 0.11)	–0.00 (–0.10 to 0.09)	–0.01 (–0.11 to 0.09)
Triglyceride, mmol/L	0.71 (0.25)	0.70 (0.26)	0.65 (0.32)	–0.00 (–0.09 to 0.09)	0.05 (–0.04 to 0.14)	0.05 (–0.04 to 0.14)

Values are shown in means (SD).

a Differences between groups are calculated as mean differences with 95% CI for continuous variables (using univariate linear regression model). Mean differences between groups adjusted for confounders (sex, age at examination, parity, pregestational BMI, smoking during pregnancy, educational level (mother), breastfeeding, birthweight (SDS), puberty onset (yes/no)) with multiple linear regression analysis.

bpm: beats per minute; FET: frozen embryo transfer; fresh-ET: fresh embryo transfer; NC: natural conception; SDS: standard deviation score.

Bold text indicates significant differences between the study groups investigated.

**Table 4.** Cardiovascular outcomes for boys included in the study divided into the three study groups and compared pairwise.

	FET n = 87	Fresh-ET n = 102	NC n = 102	FET vs fresh-ET^a^ adjusted	FET vs NC^a^ adjusted	Fresh-ET vs NC^a^ adjusted
Age at examination, years	8.46 (0.56)	8.54 (0.51)	8.50 (0.59)	–0.13 (–0.32; 0.06)	–0.09 (–0.28 to 0.11)	0.05 (–0.15 to 0.24)
Systolic blood pressure, mmHG	106.48 (9.21)	106.44 (8.11)	108.36 (8.40)	–0.21 (–3.09 to 2.67)	2.37 (–5.35 to 0.60)	–2.01 (–5.08 to 1.06)
Systolic blood pressure, SDS	0.61 (0.81)	0.57 (0.73)	0.81 (0.75)	–0.00 (–0.26 to 0.26)	–0.24 (–0.51 to 0.04)	–0.22 (–0.50 to 0.06)
Diastolic blood pressure, mmHG	63.74 (6.03)	64.73 (6.86)	64.92 (7.16)	–0.91 (–3.19 to 1.38)	–1.30 (–3.67 to 1.07)	–0.34 (–2.71 to 2.03)
Diastolic blood pressure, SDS	0.34 (0.51)	0.41 (0.58)	0.46 (0.62)	–0.07 (–0.27 to 0.13)	–0.12 (–0.32 to 0.08)	–0.05 (–0.25 to 0.16)
Heart rate, bpm	75.17 (9.53)	77.16 (9.82)	75.00 (9.71)	–1.84 (–5.12 to 1.43)	–0.26 (–3.68 to 3.16)	1.52 (–1.86 to 4.90)
Cholesterol, mmol/L	3.85 (0.62)	3.98 (0.63)	4.05 (0.66)	–0.14 (–0.34 to 0.07)	–**0.22 (**–**0.43to** –**0.00)**	–0.07 (–0.29 to 0.15)
Low-density lipoprotein, mmol/L	2.15 (0.56)	2.31 (0.56)	2.31 (0.56)	–**0.19 (**–**0.37to** –**0.00)**	–**0.20 (**–**0.39to** –**0.01)**	–0.00 (–0.14 to 0.13)
High-density lipoprotein, mmol/L	1.62 (0.34)	1.61 (0.37)	1.62 (0.38)	0.02 (–0.11 to 0.15)	0.02 (–0.12 to 0.15)	–0.01 (–0.11 to 0.10)
Triglyceride, mmol/L	0.57 (0.20)	0.65 (0.35)	0.69 (0.29)	–**0.10 (**–**0.18to** –**0.01)**	–**0.10 (**–**0.19to** –**0.01)**	–0.00 (–0.09 to 0.09)

Values are shown as mean (SD).

a Differences between groups are calculated as mean differences with 95% CI for continuous variables (using univariate linear regression model). Mean differences between groups adjusted for confounders (sex, age at examination, parity, pregestational BMI, smoking during pregnancy, educational level (mother), breastfeeding, birthweight (SDS), puberty onset (yes/no)) with multiple linear regression analysis.

bpm: beats per minute; FET: frozen embryo transfer; fresh-ET: fresh embryo transfer; NC: natural conception; SDS: standard deviation score.

Bold text indicates significant differences between the study groups investigated.

In the sensitivity analyses restricted to the pre-pubertal children, significant differences remained for SBP, SBP (SDS), and heart rate for girls born after FET compared with fresh-ET (adj. mean difference: SBP: 3.14, 95% CI (0.65 to 5.64), SBP (SDS): 0.27, 95% CI (0.04 to 0.51), heart rate: 5.41, 95% CI (1.49 to 9.33)). In the sensitivity analysis for girls, SBP was also significantly higher in girls born after FET compared with girls born after NC (adj. mean difference: SBP 2.90, 95% CI (0.37 to 5.44)). No differences were seen regarding BP and heart rate between the three groups of pre-pubertal boys (data not shown). Further adjusting the results for childhood BMI, which is known to be associated with BP, did not change any of the BP results.

For the 479 children born from 2009 to 2012, we had information on 41 cases of maternal hypertensive disorders in pregnancy. Adjusting for this factor did not change any of the BP results for either boys or girls.

An analysis of the association between birthweight and childhood SBP conducted with univariate linear regression analysis showed no significant association in our cohort of children.

### Lipid profiles

For the girls, no significant differences were found in the adjusted analyses of the lipid profiles between any of the three groups (Table 3). For the boys born after FET, cholesterol, LDL, and triglycerides were significantly lower compared with boys born after NC (adj. mean difference: cholesterol: –0.22, 95% CI (–0.43 to –0.00), LDL: –0.20, 95% CI (–0.39 to –0.01), triglyceride: –0.10, 95% CI (–0.19 to –0.01)). Further, LDL and triglycerides were lower in boys born after FET compared with fresh-ET (adj. mean difference: LDL: –0.19, 95% CI (–0.37 to –0.00), triglyceride: –0.10, 95% CI (–0.18 to –0.01)) (Table 4). Sensitivity analyses restricted to pre-pubertal children did not change the results for the lipid profiles for either sex (data not shown).

An additional sub-analysis was conducted to investigate the influence of maternal age on our results. Adding maternal age to the multiple regression analysis did not change any of our results significantly except for the boys where the difference in cholesterol and LDL between FET and NC was no longer significant (data not shown).

We did an explorative sub-analysis comparing HRT-FET (n: girls = 12, boys = 15) and NC-FET (n: girls = 78, boys = 56). After stratification on sex and conducting pairwise comparisons for SBP, SBP (SDS), DBP, DBP (SDS), heart rate, and lipid profiles, we found no significant differences for any outcomes for either girls or boys (data not shown).

## Discussion

In an analysis of BP, heart rate, and lipid profile in 606 children born after FET, fresh-ET, and NC, and stratified on sex, we found significantly higher SBP, SBP (SDS), and heart rate in girls born after FET compared with girls born after fresh-ET. Boys born after FET had a more favourable lipid profile compared with boys born after fresh-ET and NC.

Previous studies on cardiovascular health after ART show diverging results. A Nordic population-based register study reported no differences in the risk of cardiovascular disease between FET and fresh-ET, but the follow-up period was of relatively limited duration (a median of 7.5 years in the ART group and 13.6 years in the NC group) (Norrman *et al.*, 2021). Elhakeem *et al.* (2023) conducted a sub-analysis based on data from two cohorts and found similar SBP, DBP, heart rate, HDL, LDL, triglyceride, and higher cholesterol when FET and fresh-ET were compared with NC. The first study raising concern regarding cardiovascular health in children born after ART came from Switzerland where 65 children born after ART (herein 17 born after FET) and 57 controls (born after NC) were examined at 11–12 years of age. The children born after ART were found to have a thicker intima-media in the carotid arteries, impaired flow-mediated vasodilation, and increased pulse wave velocity, but no detectable differences were found between FET and fresh-ET (Scherrer *et al.*, 2012). The cohort was followed-up 5 years later where similar alterations were found indicating premature vascular ageing in the ART population. In addition, 8 individuals out of 52 (15.4%) in the ART group fulfilled the criteria of hypertension, whereas it was only 1 out of 43 (2.3%) in the NC group (Meister *et al.*, 2018). These two studies were, however, of very limited sample size with a high risk of selection bias.

In our own sub-cohort of children examined with cardiac MRI, no differences between FET, fresh-ET, and NC were found regarding stiffness of the arterial tree determined by aortic distensibility and pulse wave velocity (Mizrak *et al.*, 2022b) and without differences between pre-pubertal girls and boys (Mizrak *et al.*, 2022a). A recently published study (n = 2741 children aged 4–11 years) comparing children born after FET with fresh-ET, and adjusting for several relevant confounders, found increased SBP and metabolic sum score in children born after FET compared with fresh-ET but only in the group where the mothers were overweight, underlining the importance of parental factors (Zhang *et al.*, 2022). Further, Green *et al.* (2013) investigated lipid profiles of children aged 3.5–11 years of age born after FET (n = 42) and fresh-ET (n = 72) and found lower HDL levels in children born after FET compared with those born after NC.

Our study is the first to report long-term cardiovascular outcomes after FET versus fresh-ET stratified by sex; hence, our results are not directly comparable with the previous studies. Our finding of increased SBP in girls born after FET might be caused by the increased heart rate also found in girls born after FET. This hypothesis relies on BP being the product of cardiac output multiplied by total peripheral resistance. Further, cardiac output is the product of heart rate and stroke volume. This could only be the case given the assumption that stroke volume was similar in all three groups.

Pubertal onset is earlier in girls than boys and puberty causes an accelerated increase in BP (Kjaer *et al.*, 2022). Therefore, comparing boys and girls at the same age is challenging. In addition, studies have found that girls born after ART enter puberty earlier, and boys later, compared with their naturally conceived peers (Klemetti *et al.*, 2022). We adjusted for ‘onset of puberty’ in the main analyses and further made a sensitivity analysis excluding girls and boys who had entered puberty. In the pre-pubertal girls, the differences in SBP and heart rate between FET, fresh-ET, and NC were even more pronounced.

The prospective collection of data and the fact that all children were examined in the same setting with a small group of medically trained and blinded examinators were clear strengths of this study. Further, we had very few missing values from the clinical examinations and blood sampling and a thorough non-participant analysis showed only minor differences between participants and non-participants, which served to minimize selection bias.

In line with previous studies, we found significantly higher birthweight in the FET group compared with children born after fresh-ET and NC for both sexes (Maheshwari *et al.*, 2018). The association between birthweight and BP later in life is U-shaped (Gerber and Stern, 1999), and a recent meta-analysis revealed an association between LGA and increased childhood BP (Zhang *et al.*, 2023). This association may be caused by the well-documented association between high birthweight/LGA and obesity (Matthews *et al.*, 2017; Kapral *et al.*, 2018; Derraik *et al.*, 2020), and obesity and hypertension (Drozdz *et al.*, 2021). Even though this association was not seen in our cohort, factors such as gestational diabetes (Vlachová *et al.*, 2015; Lowe *et al.*, 2019), maternal obesity (Ohlendorf *et al.*, 2019; Voerman *et al.*, 2019), low educational level of the parents (Chaparro and Koupil, 2014; Bramsved *et al.*, 2018), and lack of breastfeeding (Yan *et al.*, 2014; Mantzorou *et al.*, 2022), which have been shown to increase the risk of obesity in the offspring, are relevant confounders in our study and are included in our adjusted analyses.

###  

A limitation to our study was the lack of adjustment for parental BP since an association between parental BP and offspring BP has previously been observed (Fuentes *et al.*, 2000; Jang *et al.*, 2023), but adjusting for hypertensive disorders in pregnancy did not change the results. Another limitation to the study was the fact that included children were conceived by ART methods used from 2009 to 2013. In the FET group, the clear majority had a cleavage-stage embryo, frozen with slow-freeze, transferred. In the fresh-ET group, 86% had a cleavage-stage embryo transferred. Today, vitrification and warming of blastocysts are the dominating FET procedures used; hence, the included children are conceived after the use of ART techniques that have been replaced by more effective methods. However, research has demonstrated that LGA and hypertensive disorders in pregnancy are equally present after vitrification of blastocysts and slow-freeze of cleavage-stage embryos (Kaartinen *et al.*, 2016; Ernstad *et al.*, 2019). This suggests that the two freezing techniques and prolonged embryo culture lead to similar adverse outcomes.

Unfortunately, we do not have information on cause of infertility for parents in our two ART groups. It is plausible to consider that individuals with PCOS, who are recognized for having a less favourable cardiometabolic risk profile, may be disproportionately represented in the FET group. This could be attributed to their propensity to yield a higher number of oocytes, thereby raising the likelihood of having embryos in the freezer.

In our study, 13.5% of the children born after FET were conceived in an HRT-FET cycle. Research has shown that HRT-FET is associated with more adverse obstetric and neonatal outcomes compared with NC-FET and modified NC-FET (Asserhøj *et al.*, 2021; Moreno-Sepulveda *et al.*, 2021; Rosalik *et al.*, 2021; Busnelli *et al.*, 2022; Roelens *et al.*, 2022). The lack of a corpus luteum in the HRT-FET is proposed to be part of the explanation for the poorer outcome after FET because of the lack of various vasoactive hormones produced by the corpus luteum, which are important for early utero-placental development (von Versen-Höynck *et al.*, 2019a, 2019b). We found no differences between HRT-FET and NC-FET for all investigated outcomes stratified on sex although the statistical power inherent in these exploratory sub-analyses was limited.

Further, it should be emphasized that all outcomes in the present study are secondary. Although we performed numerous comparisons, we decided not to perform correction for multiple testing as this would raise the likelihood of failing to detect true differences in outcomes. Hence, the observed differences in cardiovascular outcomes may be overstated and ought to be confirmed in future studies.

The disparities in long-term health outcomes observed in our study, as well as in the existing literature, may imply that the actual differences in cardiovascular health between children born after ART and NC are small. Moreover, the clinical significance of these variances could be a subject of debate, as their implications in adulthood remain uncertain, primarily owing to the relatively young age of the ART population in general and the FET population in particular.

## Conclusion

In this study where a large cohort of children born after FET, fresh-ET, and NC has been investigated, we observed elevated SBP, SBP (SDS), and heart rate among girls conceived after FET when compared with girls conceived after fresh-ET. These differences persisted in sensitivity analyses restricted to pre-pubertal girls. Notably, these disparities were not found in boys. Further, boys born after FET exhibited more favourable lipid profiles compared with boys born after fresh-ET and NC. These findings suggest that girls and boys may display different susceptibility to intrauterine perturbances affecting the cardiovascular system in childhood and this emphasizes the need for further long-term follow-up studies on children born after ART stratified on sex.

## Data Availability

The data underlying this article will be shared on reasonable request to the corresponding author.
